# Assessment of intuitive eating in Saudi Arabia and its relationship with sociodemographic factors and nutritional indicators using an Arabic version of the Intuitive Eating Scale-2

**DOI:** 10.3389/fnut.2025.1585856

**Published:** 2025-06-10

**Authors:** Eram Albajri, Manal Naseeb

**Affiliations:** Department of Clinical Nutrition, Faculty of Applied Medical Sciences, King Abdulaziz University, Jeddah, Saudi Arabia

**Keywords:** intuitive eating, BMI, Intuitive Eating Scale 2, sociodemographic factors, Saudi Arabia, Arabic

## Abstract

**Background:**

Intuitive eating (IE) is an eating style where the person responds to the bodily signals of satiety and hunger. This study assessed IE in Saudi Arabia using the newly validated Saudi Arabic Intuitive Eating Scale 2 (IES-2). It also examined the influence of sociodemographic factors, sex, and BMI on the scale and subscale scores.

**Methods:**

This cross-sectional study gathered data online, using the Saudi Arabic IES-2, from individuals aged 18 years and older with a BMI of 18.5 kg/m^2^ or higher residing in Saudi Arabia.

**Results:**

There were 387 respondents (57.9% women) with an average age of 31.83 ± 11.06 years. BMI showed a significant negative association with the total scale and subscale scores (*p* < 0.05). The total IE score was significantly influenced by age, sex, BMI, educational level, employment status, and recent weight change. Sex specifically affected the scores for the “Eating for Physical Rather than Emotional Reasons “(EPR) and “Reliance on Hunger and Satiety Cues for Eating subscales” (RHSC). Employment status was a significant predictor for the RHSC subscale score. Age and recent weight change specifically influenced the Unconditional Permission to Eat subscale score.

**Conclusion:**

Our findings emphasize the multidimensional impact of sociodemographic factors on IE behaviors among Saudis, and the importance of culturally customized interventions to promote IE practices in the region. Preliminary findings support the promotion of IE in public health and prevention initiatives. Future research should explore the longitudinal relationships and intervention strategies aimed at enhancing IE practices.

## 1 Introduction

Excess weight contributes to a variety of diseases that increase mortality rates, including type 2 diabetes and cardiovascular disease (1). The prevalence of overweight and obesity has increased dramatically worldwide. In Saudi Arabia, the overall rate in adults surpasses 60% (2), emphasizing a continuous upward trend, with obesity projected to increase from 36.8 to 42.9% by 2026 (3). Thus, prevention and treatment strategies are needed. The traditional method of food restriction and exercise may not be effective in reducing body mass long term, and often leads to weight cycling, physical and mental health problems, and the development of disordered eating behaviors (DEBs) such as emotional or uncontrolled eating, and cognitive restraint (4). Therefore, there is an urgent need for a new approach.

Intuitive eating (IE) is a new eating behavior approach based on replacing the diet mentality of, for example, skipping meals or calorie counting, with a mentality of respecting and trusting the body’s natural drive to eat. In IE, food consumption is guided by natural hunger and fullness signals, implying that one should eat when hungry and stop when satisfied. This approach is based on the idea that our bodies inherently understand the type and amount of food needed to maintain health and a healthy weight (5). As a result, individuals can trust their own body to make nutrient-dense, energy-rich, and enjoyable food choices without having to worry about external factors like mood or peer pressure. Despite its early formulation, IE received limited attention before 2006. This new approach to obesity management continues to grow in popularity in recent years, as it shifts the focus from food intake to the relationship between oneself and food (5).

A growing body of literature suggests that such approaches show improvement in DEBs, body image, emotional health, weight management, even in the absence of significant weight reduction (6, 7). Narrative Review of 2024 Comparative analysis of dietary and non-dietary randomized controlled trials revealed that whereas dietary interventions typically yielded greater initial weight loss, non-dietary strategies more reliably enhanced dietary eating behaviors and psychological effects over time. IE has been found to be related to anthropometric measurements (8–10). For example, lower body weight, reduced waist-to-hip ratio, and lower body mass index (BMI) were associated with higher IE values (4, 11). Additionally, more studies link weight maintenance with IE than with other weight loss methods (12).

A number of scales have been developed to measure IE. The Intuitive Eating Scale 2 (IES-2), created by Tylka and Kroon Van Diest (13), is the most widely used. The IES-2 has been validated as a tool to measure IE and has shown good results for internal consistency, test-retest reliability, and construct validity. Since culture, beliefs, and attitudes toward food may impact the interpretation of any eating behavior scale, the IES-2 was adapted for different cultures, across which it proved to be valid and reliable, for example, the American, French, Turkish, Lebanese, and German cultures (13–17). While the Arabic version of the Intuitive Eating Scale-2 (IES-2) has been validated in a sample of Lebanese community, confirming its reliability and construct validity, it is crucial to acknowledge the significant cultural disparities among Arabic-speaking cultures. Divergences in dietary beliefs, eating habits, and cultural attitudes toward food may affect the perception and practice of intuitive eating, even among populations who share the same language (17). Based on that, adapting the IES-2 for the Arabic-speaking population in Saudi Arabia was required (unpublished). Therefore, this study aims to assess IE in Saudi Arabia using the new validated Saudi Arabic IES-2. The study also examines associations between sociodemographic factors, sex and BMI, and the IES-2 and its subscales.

As far as we know, the current research is among few that have explored the prevalence of IE and its relationship with several factors, such as BMI, which is an understudied issue in Saudi Arabia. Thus, this research will enhance knowledge in this area and may provide insight into an alternative non-dieting solution that has shown promising outcomes.

## 2 Materials and methods

This study employed a cross-sectional and conducted over a period from May 2021 to February 2024. The extended duration of data collection was primarily due to low interest in research participation, which posed challenges in recruiting a sufficient number of participants. The study used an online survey to collect data. Advertisement through social media was used to recruit participants from the Saudi community. The study used a convenient sample. Once the participants clicked on the study’s link in the advertisement, they were required to provide informed consent before completing the survey. The survey consisted of (1) a sociodemographic section (with questions on age, sex, ethnicity, nationality, educational level, field of study, job status, marital status, region of residency in Saudi Arabia, and household income); (2) questions related to general health status, height, current weight, pregnancy, and mental health; and (3) the IES-2.

The inclusion criteria included being 18 years or older, male or female, and having a BMI of 18.5 kg/m^2^ or higher. Exclusion criteria included person who do not live in Saudi Arabia, women who are pregnant or breastfeeding, anyone with a history of bariatric surgery, upcoming surgery, strict dieting, eating disorders, mental health issues, metabolic disorders affecting eating, and persons on medications that affect eating and metabolism. The sample size was established following the guideline of 10–15 participants for each predictor variable in regression analyses (18). To achieve adequate statistical power across five models, a target sample size of around 400 participants was aimed for. The Research and Ethics Committee at King Abdulaziz University Hospital granted approval for this study (Reference no.: HA-02-J-008) in February 2021.

### 2.1 The Saudi Arabic Intuitive Eating Scale 2 (IES-2)

This study used the new Saudi Arabic version of the IES-2, translated and validated by Albajri and Naseeb (unpublished) based on the final IES-2 version by Tylka and Kroon Van Diest (13). A bilingual translator translated the scale forward, followed by a back translator for conceptual equivalence. A panel of experts reviewed the items, after which the translated version was pilot tested with 20 participants to determine its clarity and comprehension. The scale employs a 5-point Likert format, with options ranging from “Strongly disagree” to “Strongly agree.” The Saudi Arabic version maintains the 23 items of the original IES-2 that provide a total score, with higher scores indicating greater practice of IE. These items correspond to four distinct subscales: “Unconditional Permission to Eat” (UPE), which has 6 items; “Eating for Physical Rather than Emotional Reasons” (EPR), with 8 items; “Reliance on Hunger and Satiety Cues for Eating” (RHSC), with 6 items; and the “Body-Food Choice Congruence” (BFCC) subscale, with 3 items. Although the full validation study is not yet published, preliminary analyses demonstrated good to acceptable psychometric properties in a similar population. In the current sample, the translated IES-2 showed good internal consistency, with subscale Cronbach’s alpha values ranging from 0.62 to 0.87, and an overall alpha of 0.84. The slightly lower alpha observed in one subscale (α = 0.62) may be attributed to cultural or linguistic nuances affecting item interpretation, or to reduced item variability in the current sample. Such variation is not uncommon when using newly translated instruments across different populations. Overall, these findings are consistent with the original validation study by Tylka and Kroon Van Diest (13), which reported subscale alphas between 0.80 and 0.88, and an overall alpha of 0.89 (13).

### 2.2 Statistical analysis

Descriptive statistics were used to describe the data. Categorical independent variables were coded as binary (dummy) variables (1 = category present, 0 = category absent) to allow for comparison against the reference category. All tests were two-sided, with statistical significance set at *p* < 0.05.

Independent variables were chosen based on theoretical rationale, drawing from prior literature and their conceptual relevance (4, 13, 19), alongside exploratory stepwise regression. This stepwise approach applied a significance level of *p* = 0.05 for including variables and *p* = 0.10 for excluding them, enabling the identification of additional variables that significantly enhanced the model’s explanatory power. Multiple linear regression analysis was then used to test individuals’ overall mean IE score via regression against individuals’ sociodemographic characteristics, anthropometric measurements, and health-related characteristics. The associations between individuals’ predictors and their IES score were expressed as beta coefficients with their associated 95% confidence intervals. Assumptions of linear regression were tested, including normality, linearity, homoscedasticity, multicollinearity, and independence of residuals. IBM SPSS Statistics Version 27.0 (Armonk, NY: IBM Corp) was used for the analyses.

## 3 Results

A total of 559 individuals initially responded to the survey. However, only 387 participants (57.9%, *n* = 224 women; 42.1%, *n* = 163 men) were included in the final dataset after applying inclusion criteria and data cleaning procedures. This final sample (*n* = 387) was slightly below the targeted sample size of 400 participants. The mean participant age was 31.83 ± 11.06 years, while the mean BMI was 25.12 ± 5.40 kg/m^2^. Most of the respondents (92.8%, *n* = 359) were Saudis; 52.7% (*n* = 204) were single. The majority of the respondents (87.8%, *n* = 202) held a diploma or higher educational degree. Two-thirds of the respondents were employed (67.4%, *n* = 261), with most earning less than 5000 Saudi Riyals (39.3%, *n* = 152) or within the range of 5000 and 15000 Saudi Riyals (33.3%, *n* = 129) Over two-thirds of the respondents (71.8%, *n* = 278) resided in the Western region of Saudi Arabia. See Table 1 for full details.

**TABLE 1:** Participants’ sociodemographic characteristics.

Demographic characteristic	Frequency	Percentage
**Sex**		
Male	163	42.1
Female	224	57.9
Age (years), mean (SD)	31.83 (11.06)	
**Nationality**		
Non-Saudi	28	7.2
Saudi	359	92.8
**Marital status**		
Single	204	52.7
Married	155	40.1
Divorced or widowed	28	7.2
Body weight (kg), mean (*SD*)	68.59 (17.37)	
Body mass index (kg/m^2^), mean (*SD*)	25.12 (5.40)	
**Weight change within 3 months?**		
Yes	116	30.0
No	271	70.0
Educational level		
High school or lower	47	12.1
Diploma	202	52.2
Bachelor degree	31	8.0
Graduate degree	107	27.7
**Employment status**		
Unemployed	126	32.6
Employed	261	67.4
**Monthly household income (HHI) (Saudi Riyal/month)**		
<5000	152	39.3
5000–15000	129	33.3
16000–30000	73	18.9
>30000	33	8.5
**Region of residence**		
Northern	19	4.9
Southern	37	9.6
Eastern	26	6.7
Central	27	7.0
Western	278	71.8
Total IES-2 score, mean (*SD*)	3.43 (0.53)	
**Subscale scores, mean (*SD*)**		
UPE	3.12 (0.67)	
EPR	3.61 (0.83)	
RHSC	3.46 (0.83)	
BFCC	3.54 (0.91)	

IES-2, Intuitive Eating Scale 2; UPE, Unconditional Permission to Eat; EPR, Eating for Physical Rather than Emotional Reasons; RHSC, Reliance on Hunger and Satiety Cues for Eating; BFCC, Body-Food Choice Congruence; SD, standard deviation. Data are presented as numbers (frequency, percentage).

The mean scores for the IES-2 and its subscales are shown in Table 1, while Table 2 displays the results of the multiple linear regression analysis. The mean total IE score for the sample was 3.43 ± 0.53. The regression model for the total IE score explained 18% of the variance and identified that sex, BMI, employment status, weight change, and educational level as significant predictors (R^2^ = 0.18, *F*(7, 379) = 11.89, *p* < 0.001). Women scored 0.14 points higher than men (*B* = 0.14, 95% CI: 0.04–0.25, *p* = 0.008). BMI had a significant negative relationship with the total IE score (*B* = −0.02, 95% CI: −0.03, −0.01, *p* < 0.001): for every one-unit increase in BMI, the total score decreased by 0.02. Individuals who were employed scored lower (by 0.20) compared with those who were unemployed (*B* = −0.20, 95% CI: −0.31, −0.08, *p* < 0.001). Participants who reported losing weight in the last 3 months showed lower total IE scores (by 16) score (*B* = −0.16, 95% CI: −0.27, −0.05, *p* = 0.005). Among educational levels, only having a graduate degree had a positive impact on the total IE score (*B* = 0.24, 95% CI: 0.07, 0.41, *p* = 0.007).

**TABLE 2:** Multiple linear regression analysis of the Intuitive Eating Scale-2 and subscale scores.

Scale	Variable	*B*	SE_b_	β	*p*-value	95% CI	
						**LL**	**UP**
**Total IE score**							
	(Constant)	3.91	0.14		<0.001*	3.63	4.19
	Sex (vs. men)	0.14	0.05	0.13	0.008	0.04	0.25
	BMI	−0.02	0.00	−0.21	<0.001*	−0.03	−0.01
	Employment (vs. unemployed)	−0.20	0.06	−0.18	<0.001*	−0.31	−0.08
	Weight change (vs. none)	−0.16	0.06	−0.14	0.005*	−0.27	−0.05
	Educational level (vs. high school)						
	Diploma	0.11	0.08	0.10	0.182	−0.05	0.26
	Undergraduate degree	0.04	0.11	0.02	0.693	−0.18	0.27
	Graduate degree	0.24	0.09	0.20	0.007*	0.07	0.41
**UPE**							
	(Constant)	3.94	0.17		<0.001*	3.59	4.28
	Sex (vs. men)	−0.04	0.07	−0.03	0.522	−0.17	0.09
	BMI	−0.02	0.01	−0.14	0.008*	−0.03	0.00
	Age	−0.01	0.00	−0.12	0.015*	−0.01	0.00
	Weight change (vs. none)	−0.41	0.07	−0.28	<0.001*	−0.55	−0.26
**EPR**							
	(Constant)	4.26	0.23		<0.001*	3.80	4.71
	Sex (vs. men)	0.29	0.09	0.17	<0.001*	0.12	0.46
	BMI	−0.03	0.01	−0.20	<0.001*	−0.05	−0.02
	Educational level (vs. high school)						
	Diploma	−0.16	0.13	−0.09	0.235	−0.41	0.10
	Undergraduate degree	−0.20	0.18	−0.06	0.285	−0.56	0.17
	Graduate degree	0.16	0.14	0.09	0.251	−0.11	0.44
**RHSC**							
	(Constant)	4.11	0.21		<0.001*	3.69	4.53
	Sex (vs. men)	0.20	0.09	0.12	0.023*	−0.03	0.36
	BMI	−0.02	0.01	−0.16	0.002*	−0.04	−0.01
	Employment (vs. unemployed)	−0.24	0.09	−0.13	0.010*	−0.41	−0.06
**BFCC**							
	(Constant)	3.61	0.29		<0.001*	3.03	4.19
	Sex (vs. men)	0.26	0.10	0.14	0.009*	0.07	0.45
	BMI	0.00	0.01	0.02	0.640	−0.01	0.02
	Nationality (vs. non-Saudi)	−0.38	0.17	−0.11	0.013*	−0.72	−0.04
	Educational level (vs. high school)						
	Diploma	0.24	0.14	0.13	0.099	−0.04	0.52
	Undergraduate degree	0.17	0.20	0.05	0.405	−0.23	0.57
	Graduate degree	0.46	0.16	0.23	0.005*	0.14	0.77
	Income (vs. <5000)						
	5000–15000	−0.40	0.11	−0.21	<0.001*	−0.62	−0.17
	16000–30000	−032	0.14	−0.14	0.018*	−0.59	−0.06
	>30000	−0.52	0.18	−0.16	0.005*	−0.88	−0.16

BFCC, Body-Food Choice Congruence; BMI, body mass index; CI, confidence interval; EPR, Eating for Physical Rather than Emotional Reasons; IE, intuitive eating; LL, lower bound; RHSC, Reliance on Hunger and Satiety Cues; SEb, standard error for the unstandardized B; UP, upper bound; UPE, Unconditional Permission to Eat; B, unstandardized beta coefficient; β, Standardized beta coefficient. Reference variable for each category: men for sex; unemployed for employment; non-Saudi for nationality, none changed for weight loss; high school for educational level; income of <5000 for income.

*Statistical significance set at 0.05. Model Statistics: Total IE Score Model: R^2^ = 0.18, adj R^2^ = 0.175, F(7, 379) = 11.89, *p* < 0.001. UPE Subscale Score Model: R^2^ = 0.143, adj R^2^ = 0.134, F(4, 382) = 15.98, *p* < 0.001. EPR Subscale Score Model: R^2^ = 0.103, adj R^2^ = 0.091, F(5, 381) = 8.755, *p* < 0.001. RHSC Subscale Score Model: R^2^ = 0.081, adj R^2^ = 0.074, F(3, 383) = 11.23, *p* < 0.001. BFCC Subscale Score Model: R^2^ = 0.103, adj R^2^ = 0.081, F(9, 377) = 4.792, *p* < 0.001.

As Table 2 shows, the UPE score for the overall sample was 3.12 ± 0.67. The UPE score model explained 14.3% of the variance and showed that BMI, age, and weight change were significant predictors in the model (R^2^ = 0.143, F(4, 382) = 15.98, *p* < 0.001). BMI and age showed a significant negative relationship with the UPE score. With each one-unit increase in BMI, the UPE score decreased by 0.02 (*B* = −0.02, 95% CI: −0.03, 0.00, *p* < 0.008), and for each one-unit increase in age, the UPE score decreased by 0.01 (*B* = −0.01, 95% CI: −0.01, 0.00, *p* < 0.015). As for weight, participants who lost weight had a lower UPE score by 0.41. Sex has no significant effect on UPE score (*p* > 0.05).

The mean EPR score was 3.61 ± 0.83. The EPR score model explained 10.3% of the variance. Sex and BMI were significant predictors for the EPR score (R^2^ = 0.103, F(5, 381) = 8.755, *p* < 0.001). Scores were higher by 0.29 units for women, and BMI showed a significant negative relationship with the EPR score (*B* = −0.03, 95% CI: −0.05, −0.02, *p* < 0.001): for each one-unit increase in BMI, the EPR score decreased by 0.03. Education had no significant effect.

The RHSC score for the overall sample was 3.46 ± 0.83. The score of the RHSC subscale was predicted by sex and employment status (R^2^ = 0.081, F(3, 383) = 11.23, *p* < 0.001). Women scored had higher than men by 0.20 points (*B* = 0.20, 95% CI: 0.03, 0.36, p = 0.023), and BMI had a significant negative relationship with the RHSC score (*B* = −0.02, 95% CI: −0.04, −0.01, *p* = 0.002): with each one-unit increase in BMI, the RHSC score decreased by 0.02. Participants who were employed scored lower on the RHSC subscale by 0.24 points compared with those who were unemployed (*B* = −0.24, 95% CI: −0.41, −0.06, *p* = 0.01).

The BFCC score for the overall sample was 3.54 ± 0.91, and its model explained 10.3% of the variance. Sex, nationality, education, and income were significant predictors for the BFCC score (R^2^ = 0.103, F(9, 377) = 4.792, *p* < 0.001). Women scored higher than men by 0.26 points (*B* = 0.26, 95% CI: 0.07, 0.45, *p* = 0.009), and Saudis scored lower by 0.38 points compared with non-Saudis (*B* = −0.38, 95% CI: −0.72, −0.04, *p* = 0.031). Having a graduate degree had a positive impact on the BFCC score (*B* = 0.46, 95% CI: 0.14, 0.77, *p* = 0.005). All income levels had a significantly negative impact on the BFCC score (*B* = 0.46, 95% CI: 0.14, 0.77, *p* = 0.005).

## 4 Discussion

While IE practices are gaining popularity in Western nations, they are not yet widely used in Arab countries. To the best of our knowledge, this study is the first to explore IE aspects in relation to sociodemographic characteristics in a general sample from Saudi Arabia using the Saudi Arabic IES-2. Few variations in the IES-2 and its subscales were observed. Sex, BMI, employment status, recent weight change, and educational level were significant predictors of the total IE score. Women had higher total IE, EPR, RHSC, and BFCC scores compared with men. There was a significant negative relationship between BMI and total IE, UPE, EPR, and RHSC scores. However, age was only negatively correlated with the UPE score. Focusing on recent weight change, we found that those who had recently lost weight had lower total IE and UPE scores. Total IE and RHSC scores were low among individuals who were working compared with those who were not. Having a graduate degree was positively linked to total IE and BFCC scores. BFCC scores were higher among Saudi participants compared with non-Saudi participants, and the score was negatively associated with income.

As noted in the results, the total IE score showed a negative association with BMI. This may suggest that persons with a higher BMI might be less likely to eat intuitively, which may be attributed to a history of dieting or exposure to stigma related to weight. This might impair the person’s reliance on hunger and satiety cues (4). Similar results have been reported in French and Swiss studies (14, 20). Surprisingly, we found that women recorded higher total IE scores. This finding is concordant with results of a study by Albajri and Naseeb (21), in the same culture, who observed that women consistently showed higher scores. However, studies in other populations found that women typically scored lower total IE scores compared with men (13–15). Possible explanations for these contradicting results are cultural differences, a high prevalence of emotional eating, and higher societal pressure related to body image among women in some cultures (22–25). Social and cultural norms in Saudi Arabia may influence gender differences, where women frequently experience greater pressure to meet appearance standards. This heightened pressure could lead to increased body awareness and a stronger inclination toward non-diet approaches like intuitive eating. In the present study, higher educational levels were positively linked to IE, indicating that people with advanced education may have a greater understanding of nutrition, health, and general wellness that may help them make decisions aligned with their health goals and bodily needs (4, 24). These findings highlight the importance of considering sociocultural dynamics in shaping eating behaviors within the Saudi context. Additionally, employed individuals showed lower total IE scores. This may be a result of a stressful work environment in Saudi Arabia, time constraints, and exposure to irregular eating schedules or the availability of convenience foods. Such factors may trigger emotional eating and can interfere with a person’s ability to listen to and respond to the internal signals of hunger and fullness (26). We observed that participants who lost weight in the last 3 months had lower total IE scores. This may be a result of following a structured diet or restrictive eating pattern, because by doing so the individual is relying on external regulation for their eating behavior, which would interfere with IE skills (27).

In previous reports (4, 28), women scored lower on the RHSC subscale, contrary to our results. These discrepancies may be attributed to cultural differences across populations and different social pressures around body image, which disrupt a person’s ability to rely on internal hunger and satiety cues. In the current study, BMI was inversely associated with the RHSC score, similar to results reported in other studies (13, 29, 30). As explained above, this could reflect the influence of repeated dieting or restrictive eating patterns, which could desensitize the individual to hunger and satiety cues over time. Employment status showed a similar correlation with the RHSC score, which may relate to a stressful environment, as mentioned above. Using strategies that promote mindful eating and addressing work-related issues may help mitigate the negative impact of employment on IE.

BMI and sex both demonstrated a similar negative association with the EPR subscale. Women scored higher than men on this subscale, contrary to other reports (13, 29, 30), probably due to different social norms. Higher BMIs were linked to lower scores on the EPR, likely due to emotional eating patterns brought on by societal stigmatization and diet practices (30).

The BFCC subscale was significantly associated with sex, educational level, income, and nationality. Women scored higher than men on this subscale, in contrast to other reports (13–15), possibly due to the different cultural and eating habits in women of different cultures (25). Similarly, the level of education was positively associated with the BFCC subscale, possibly due to the effect of greater knowledge on decision-making regarding food choices, as mentioned earlier (4, 24, 31). Nationality and income appeared to be associated with the BFCC subscale. The impact of nationality and culture on IE is intricate and multidimensional because cultural norms around food, body image, and emotional regulation profoundly influence eating behaviors. For instant, participants from cultures that value holistic health and balance, such as some Eastern or indigenous cultures, scored higher on this subscale, indicating a stronger alignment between food choices and personal health and body goals in those cultures (8). Surprisingly, we found that Saudis scored higher compared with participants of other nationalities. Lastly, income had a negative correlation with the BFCC score, which could be explained by the level of income. Individuals of lower income may face financial obstacles and access constraints, whereas higher-income individuals may contend with social and cultural pressures, which ultimately prevent the practice of IE (23, 32).

BMI had a negative association with the UPE subscale, similar to results reported in large French and Swiss studies (20, 29). These studies confirmed that higher permission scores correlated with lower BMI. Thus, people with higher BMIs may suffer more with Unconditional Permission to Eat because of societal stigmatization, dieting practices, and emotional eating behaviors. Moreover, we observed lower UPE scores in participants who reported losing weight in the last 3 months. This might reflect that these individuals followed structured diets (i.e., external controls) to control their food intake. This external regulation of eating behaviors interferes with IE skills. Another possible explanation is that people who lose weight through traditional ways tend to categorize food as “bad” or “good,” which interferes with the goal of the UPE subscale (27). The traditional ways of losing weight typically override the internal signals for hunger and satiety (33). However, in several studies (12, 25, 34), IE correlated with greater weight stability. Lastly, our findings were consistent with those documented by Denny et al. (24), who reported that young adults need to trust their body and eat when feeling hungry. These findings provide evidence that age negatively correlated with UPE. In other words, older individuals may be less likely to grant themselves the freedom to eat without limits.

### 4.1 Limitations, strengths, and future studies

To our knowledge, the current study is the first to examine aspects of IE in relation to sociodemographic factors in a general sample from Saudi Arabia. Among the strengths of this study is the inclusion of a large, diverse group of participants from a variety of sociodemographic backgrounds, which enhances generalizability of the findings. The inclusion of both men and women is another strength, as most published IE studies are focused on women (4, 35). The study explored many sociodemographic factors (such as age, sex, BMI, employment status, education, income, nationality, and weight change), offering a detailed comprehension of their impact on IE practices. In addition, by validating the Saudi Arabic version of the IES-2 among the Saudi population, the study helps to ensure its cultural and linguistic appropriateness. However, future studies are required to test the scale in different settings and different regions of the country. Nevertheless, there are several limitations to address. This was a preliminary investigation limited in its design and in how participants. The correlational, cross-sectional design restricts the study’s capacity to determine causal correlations between the studied variables and IE practices. Utilizing online recruitment and convenience sampling may lead to selection bias, particularly by overrepresenting individuals with internet access and a passion for health topics, which could impede the findings’ applicability to the entire Saudi population. Also, the reliance on a self-reported measure for IE, weight, height, and sociodemographic variables invites the possibility of the introduction of bias, including recall and social desirability bias, which can influence data precision. Moreover, with 71.8% of respondents hailing from the Western region, the findings may not be generalizable to the culturally and socioeconomically varied regions of Saudi Arabia. Additionally, a modest R2 values of 0.08–0.18 suggest that intuitive eating is influenced by unmeasured psychosocial and environmental factors, which underscoring the need for further research to contextualize the findings. Lastly, although validated instruments were employed, the dependence on self-reported mental health status and medication use, lacking clinical validation, might compromise the internal validity of the results and their interpretation. Subsequent studies should focus on overcoming these limitations by incorporating objective measurements, utilizing longitudinal designs, and including a wider range of geographically diverse samples through effective sampling techniques. Furthermore, investigating the connection between intuitive eating and personality traits could reveal which populations are most open to targeted interventions. Analyzing the relevance of intuitive eating in clinical settings, particularly for individuals with obesity in Saudi Arabia, might also contribute to culturally appropriate health strategies.

Data from this study can be used as a baseline for future, more comprehensive studies. However, these results offer early evidence for the promotion of IE in public and preventative health settings. More research is required to assess how IE-based interventions affect eating habits and to investigate the interactions among various components of IE that may influence these habits. It is also imperative to explore the connection between IE and psychological functioning in young, middle-aged, and older men and women.

## 5 Conclusion

The findings of this study offer important insights into intuitive eating behaviors within the Saudi population, highlighting notable correlations with sociodemographic characteristics, sex and BMI. These findings highlight the intricate interaction of cultural, physiological, and socioeconomic factors in influencing intuitive eating, underscoring the necessity for culturally informed strategies to promote healthy eating behaviors in Saudi Arabia. Future research should investigate longitudinal patterns and intervention strategies to enhance IE behaviors in this as well as similar populations.

## Data Availability

The raw data supporting the conclusions of this article will be made available by the authors, without undue reservation.
